# YOLOv11-Pose-Based Tracking Reveals Neuropeptide F Regulation of Agonistic Behavior in *Macrobrachium rosenbergii*

**DOI:** 10.3390/ani16203307

**Published:** 2026-10-20

**Authors:** Bangping Hu, Chengyuan Peng, Xiaojia Liao, Linwei Yang, Hongliang Zuo, Shaoping Weng, Jianguo He, Xiaopeng Xu

**Affiliations:** 1 State Key Laboratory of Biocontrol, Southern Marine Science and Engineering Guangdong Laboratory (Zhuhai), China-ASEAN Belt and Road Joint Laboratory on Mariculture Technology, School of Life Sciences, Sun Yat-sen University, Guangzhou 510275, China; jankos2001@163.com (B.H.); linnaa2003@163.com (X.L.); zhlzuo@163.com (H.Z.); lsswsp@mail.sysu.edu.cn (S.W.); lsshjg@mail.sysu.edu.cn (J.H.); 2 Guangdong Province Key Laboratory of Aquatic Economic Animals, Institute of Aquatic Economic Animals, Sun Yat-sen University, Guangzhou 510275, China; 3 State Key Laboratory of Mariculture Breeding, Key Laboratory of Marine Biotechnology of Fujian Province, College of Marine Sciences, Fujian Agriculture and Forestry University, Fuzhou 350002, China; pengchengyuan@stu.fafu.edu.cn (C.P.); yanglinwei@fafu.edu.cn (L.Y.)

**Keywords:** *Macrobrachium rosenbergii*, YOLOv11-pose, agonistic behavior, neuropeptide F, energy metabolism

## Abstract

The giant freshwater prawn *Macrobrachium rosenbergii* is inherently aggressive, and this innate trait constitutes a major bottleneck restricting high-density intensive aquaculture. In this study, we developed behavioral phenotyping analysis software integrating YOLOv11-pose and the SORT tracking algorithm to automatically recognize and quantify agonistic behaviors. To validate the reliability of this platform, we used it to investigate the role of neuropeptide F (NPF) through loss- and gain-of-function strategies. The platform successfully captured behavioral differences across treatments and generated results that were consistent with manual observations, confirming its accuracy and utility for automated behavioral analysis. Moreover, it provided additional kinematic metrics that are not accessible through manual observation, offering more comprehensive behavioral quantification. Mechanistically, NPF silencing reduced feed intake, suppressed growth, and disrupted hepatopancreatic glycolysis, suggesting that NPF may modulate aggression at least partly through energy metabolic pathways. Collectively, this study provides a robust tool for behavioral phenotyping in crustaceans, with potential applications for behavior-based management in intensive farming systems.

## Simple Summary

The giant freshwater prawn is naturally aggressive, and this behavior often leads to injuries and growth problems when the animals are raised in crowded farm ponds. In this study, we developed a computer-based system that uses a technique called YOLOv11-pose to automatically recognize and measure fighting behavior in prawns from video recordings. To test whether this system works reliably, we used it to study the role of a protein called neuropeptide F (NPF) in regulating aggression. The system successfully detected all the behavioral changes caused by NPF, and the results matched what human observers could see. The system also measured movement details that are too fine for the human eye to catch. Our findings show that NPF controls fighting behavior by influencing how the prawns use energy from food. This automated system provides a powerful tool for investigating the mechanisms underlying agonistic behavior in crustaceans.

## 1. Introduction

The giant freshwater prawn, *Macrobrachium rosenbergii*, is a large decapod crustacean native to Southeast Asia [1,2]. In nature, individuals frequently engage in aggressive interactions over resources such as food, shelter, and mates [3,4]. At present, *M. rosenbergii* is widely farmed as a crustacean species of high commercial value [5,6]. High-density intensive aquaculture represents the current trend in the development of its farming industry [5]. However, conflicts between individuals can lead to physical injury, marked growth disparity and increased mortality rates in *M. rosenbergii*, ultimately undermining population stability and reducing economic returns [5,7]. At present, our understanding remains limited regarding how these behavioral phenotypes manifest dynamically during real-time interactions among individuals of this species, as well as the regulatory mechanisms underlying their variation. Therefore, the development of objective, automated methods for behavioral recognition and individual classification is crucial for understanding the dynamic processes of aggressive behavior and for achieving precision management in prawn aquaculture.

Agonistic behavior in crustaceans is shaped by a complex interplay between intrinsic factors (e.g., body size and internal physiological state) and extrinsic environmental stimuli [8,9]. Descriptive ethograms have long been used to characterize fighting sequences in decapods. However, current approaches rely on manual definitions and suffer from several limitations, including high labor intensity, observer bias, and difficulty in capturing the fine-scale temporal dynamics of behavioral exchanges. For example, the strategy of Pintor et al. required observers to continuously record and score aggressive interactions in crayfish, while some commercial software packages can track only the geometric center or body contour and rely on manually triggered events, lacking deep-learning adaptations specifically designed for crustaceans [10].

Deep-learning-based approaches have been widely applied to animal behavior analysis. For instance, studies have validated multi-object tracking frameworks on *Drosophila* [11], applied attention-integrated YOLOv11 for predator detection in stingless bee protection [12], and developed a three-stage pipeline for segmenting, tracking, and classifying leaf-cutter ant behavior [13]. These methods mainly fall into two categories: object detection (including multi-object tracking) and video-based behavior recognition/classification. The former relies primarily on CNN and ViT architectures, with some approaches also employing graph networks for cross-modal feature fusion. CNN-based methods have also been applied to shrimp morphometrics and behavior analysis, with studies using keypoint localization and Detectron2-based regression for body measurement and weight estimation [14,15]. In contrast, ViT-based architectures, such as the RGBD-based ViT-Pose system, have achieved high-precision keypoint localization in *Litopenaeus vannamei*, overcoming the limitations of CNN-based local feature modeling [16]. For fighting behavior recognition, Sun et al. used DeepLabCut with a ResNet backbone to track keypoints during agonistic interactions in the Chinese mitten crab, demonstrating the potential of CNN-based feature construction for crustacean behavioral analysis [17]. However, we found that this approach transferred poorly to *M. rosenbergii*: its slender, highly variable chelipeds provide sparse semantic information around keypoints, and with limited annotated data the model failed to learn discriminative features, leading to unstable localization and poor generalization. For video-based behavior recognition, representative terrestrial studies include the PanAf20K dataset constructed by Brookes et al. (2024) for wild ape detection and behavior recognition, and the ARTEMIS multimodal framework proposed by Fazzari et al. (2025), which integrates video, image, and text modalities and achieved an mAP of 79.82 on the Animal Kingdom dataset [18,19]. However, these methods still rely heavily on large-scale videos and substantial computing power, with limited data and research on aquatic organisms, especially crustaceans.

In this study, we constructed a dual-target tracking platform based on YOLOv11s-Pose combined with handcrafted behavioral rules, specifically tailored to the slender and highly variable chelipeds of *M. rosenbergii*, to automate the quantification of agonistic behavior. The platform incorporates a handcrafted geometric rule based on cheliped keypoint intersection for detecting grappling-type fights. Using this platform, we validated its reliability through NPF loss- and gain-of-function experiments against manual observation, and further revealed the regulatory role of neuropeptide F (NPF) in complex agonistic behavior, linking NPF to feeding, growth, and glycolysis. Moreover, the platform proved to be a robust, high-throughput, and unbiased tool for capturing subtle behavioral changes in aquatic animals, offering a promising alternative to traditional labor-intensive manual observation.

## 2. Materials and Methods

### 2.1. Animals and Data Acquisition

**Figure 1:**
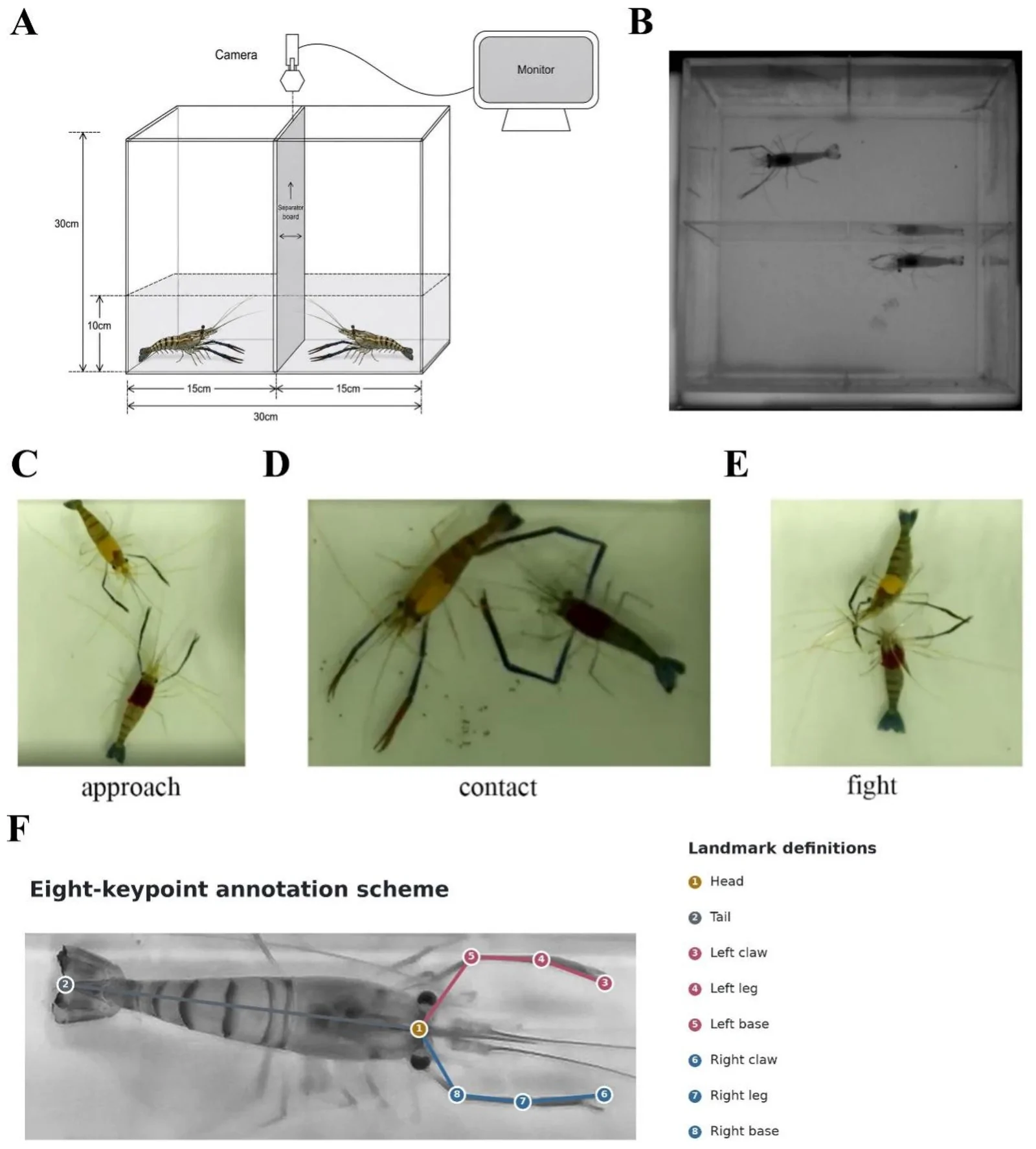
Observation apparatus and behavioral annotation for prawn fighting. (**A**) Overall schematic of the observation apparatus (generated using Chatgpt Images 2.0); (**B**) top-down view of the apparatus; (**C**) approach behavior; (**D**) contact behavior; (**E**) fighting behavior; (**F**) eight-keypoint annotation scheme for a prawn in a grayscale top-view image. The landmark set comprises the head (1), tail (2), left claw tip (3), left claw middle (4), left claw base (5), right claw tip (6), right claw middle (7), and right claw base (8). The connecting lines delineate the main body axis and the ordered landmark chains of the left and right appendages.

*M. rosenbergii* (~10 g) sourced from an aquaculture farm in Foshan, China, were acclimated in circulating freshwater at 27 ± 1 °C for at least 2 d, then randomly assigned to groups, with individuals housed separately and fed at fixed times daily. Behavioral videos were recorded in a controlled indoor arena (Figure 1A). A camera was mounted vertically above the experimental tank to capture top-down footage, ensuring complete documentation of the spatial positions and behavioral dynamics of the individuals. Videos were recorded at multiple resolutions and frame rates, such as 1080p and 720p, to clearly capture rapid movements and fighting events. For each trial, two healthy, experimentally naïve individuals were placed on opposite sides of a transparent tank (water depth: 10 cm) and separated by an opaque divider (Figure 1B). After a 20 min acclimation period, the divider was removed to allow free interaction, and the ensuing agonistic behavior was continuously recorded using the high-definition camera. Four categories of behaviors in the footage were analyzed: *none*, *approach*, *contact*, and *fight*. The definitions of these four behaviors are provided below. It should be noted that two sets of behavioral definitions are adopted in this paper. One is manual visual judgment, and the other is software-based judgment of behaviors and kinematic metrics implemented in Section 2.2 and Section 3.3.

The *none* category refers to the absence of obvious behavioral interaction between individuals, with the animals merely remaining stationary or moving freely. *Approach* is defined as a state in which the chelipeds of two individuals come within a clear contact range but do not actually make physical contact (Figure 1C). *Contact* denotes that the cheliped of at least one shrimp has made physical contact with the other individual (Figure 1D). *Fight* is defined as at least one individual using its chelipeds to perform aggressive actions against the opponent, including striking the opponent’s cephalothorax, grasping and pulling the opponent’s body or chelipeds (Figure 1E). Each fight episode is considered to end when the two individuals finally separate from each other. After rigorous frame-by-frame screening, a total of 499 high-quality original images were obtained, named the D499 raw dataset. The 499 original images of the D499 dataset were divided into three subsets of 410, 42 and 47 images. The augmented copies of each frame were assigned to the same subset as their original image without cross-subset leakage. Using the LabelMe (version 5.11.3) annotation tool, eight body keypoints of each *M. rosenbergii* individual were manually annotated in each image. Five data augmentation transformations were applied: horizontal flip, histogram equalization, Gaussian noise (σ = 8.0), clockwise rotation by 90°, and clockwise rotation by 270°. For each augmentation method, the original keypoint coordinates were retained, finally resulting in a dataset containing 2994 multi-scale images, named the D8 dataset. The D8 dataset comprises a total of 5988 prawn instance annotations, with a total of 47,904 annotated keypoints.

### 2.2. Behavior Definition

To enable automated recognition of fighting events, we decomposed the key body structures involved in *M. rosenbergii* agonistic interactions into a set of definable skeletal features, which served as the basis for the software’s behavioral classification. As shown in Figure 1F, a total of eight keypoints were selected, forming seven edges. Pixel-to-length calibration was conducted using a standard calibration object. The conversion factor is defined as follows:

$$r=\frac{1\;\mathrm{cm}}{p\;\mathrm{pixels}}$$

The r is the conversion factor, and *p* is the number of pixels corresponding to 1 cm.

The left and right claws of each prawn each consist of three keypoints (tip, middle, base), which are sequentially connected to form four claw-skeleton line segments: left claw tip-middle, left claw middle-base, right claw tip-middle, right claw middle-base. Denote these four line segments as *L*_1_, *L*_2_, *L*_3_, *L*_4_. For two different prawns with distinct IDs in the same frame, if $L_{i}^{\left(1\right)}\cap L_{j}^{\left(2\right)}\neq \emptyset$, it is recorded as a fight. A segment of consecutive frames where fighting is detected, lasting more than 10 frames, is considered a valid fighting episode. If the time interval between two valid episodes is less than 30 s, they are merged into a single episode; the merged episode is regarded as one fighting bout, i.e., one fight.

### 2.3. Model Training Configuration

Based on YOLOv11-pose, an analytical model for the interactive behaviors of paired crustacean individuals was established. The compiler versions are Python 3.11.14, PyTorch 2.12.0, and CUDA 12.8. The graphical user interface is built with PySide6 (Qt 6). The parameter settings are shown in Table 1. In this study, 200 epochs are selected based on the experimental results to prevent the model from overfitting or underfitting. The batch size is 16, which fully utilizes the GPU memory and its parallel computing capability. The remaining settings are coordinated with the optimizer to ensure that the model converges adequately.

### 2.4. Tracking Framework Based on Yolo11s-Pose

**Figure 2:**
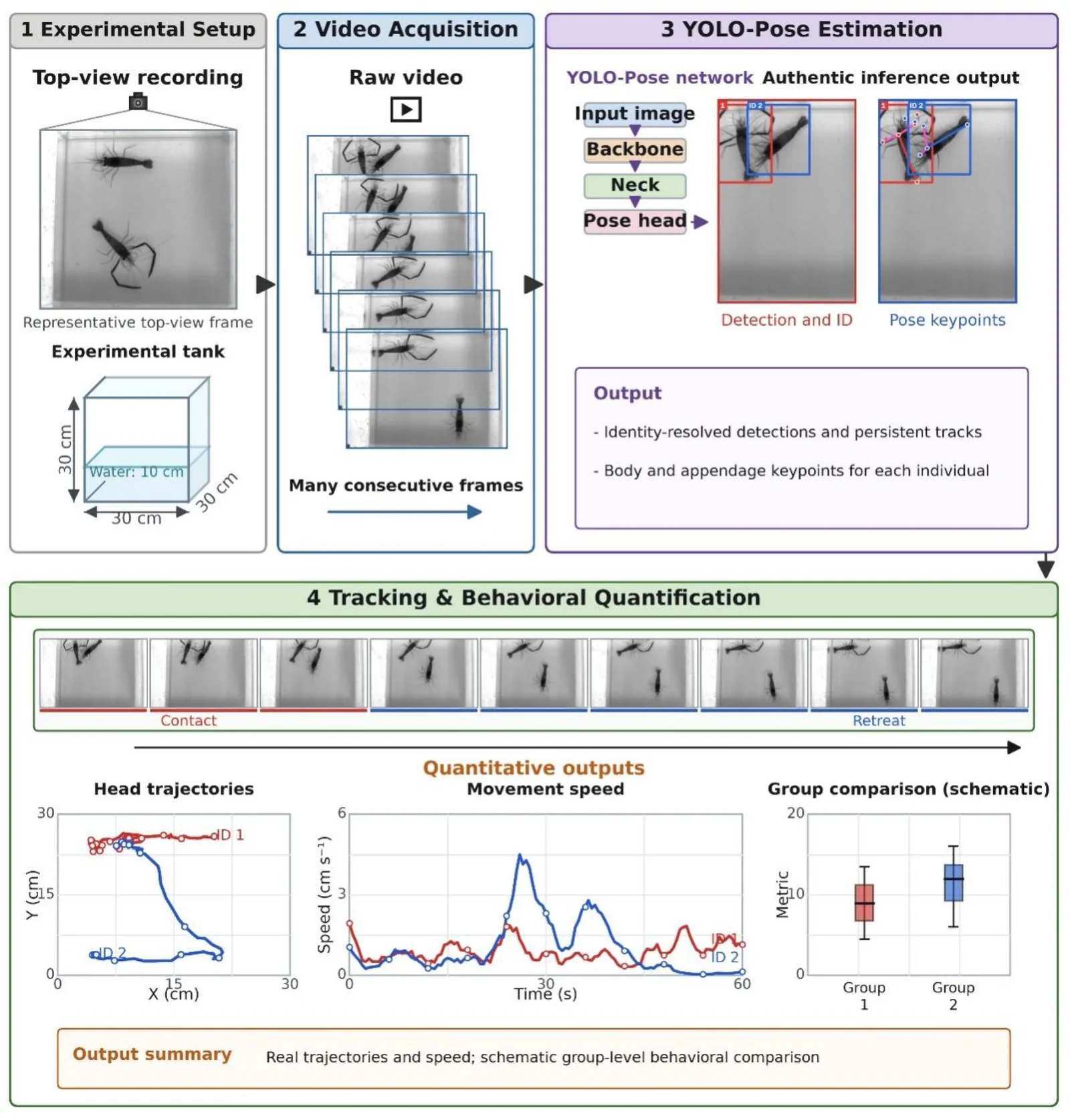
Workflow for automated analysis of dyadic prawn behavior. Top-view videos were recorded in a 30 × 30 × 30 cm experimental tank with a water depth of 10 cm. Consecutive frames were processed using YOLO-Pose to identify the two individuals and estimate their body and appendage keypoints. The identity-resolved trajectories and pose information were subsequently used to recognize behavioral transitions (e.g., contact followed by retreat) and to calculate head trajectories and movement speed.

To achieve accurate quantitative analysis of the interactive behaviors of two *M. rosenbergii* individuals, YOLOv11-Pose was selected as the core pose estimation model. The tracking pipeline is shown in Figure 2. In the implemented system, the SORT algorithm is directly adopted for dual-target tracking. As the task scenario is simple, SORT can output stable dual-target time-series data to provide reliable input for subsequent analysis. During online inference, the software collects complete frame-by-frame temporal tracking data of the two prawns. After applying calibration parameters and scale conversion, fighting events are identified based on handcrafted features, and kinematic metrics are obtained based on kinematic thresholds. This module supports visual playback, manual verification, and annotation of all behavioral segments, and enables batch export of various quantitative metrics. To accelerate analysis, TensorRT FP16 half-precision inference is deployed on a physical GPU, with batch sizes of 1, 2, 4, 8, and 16 samples supported (default: 8). Offline processing achieves an average speed of 262 FPS, fully satisfying the requirement for rapid high-throughput parsing of large-scale sample batches.

**Figure 3:**
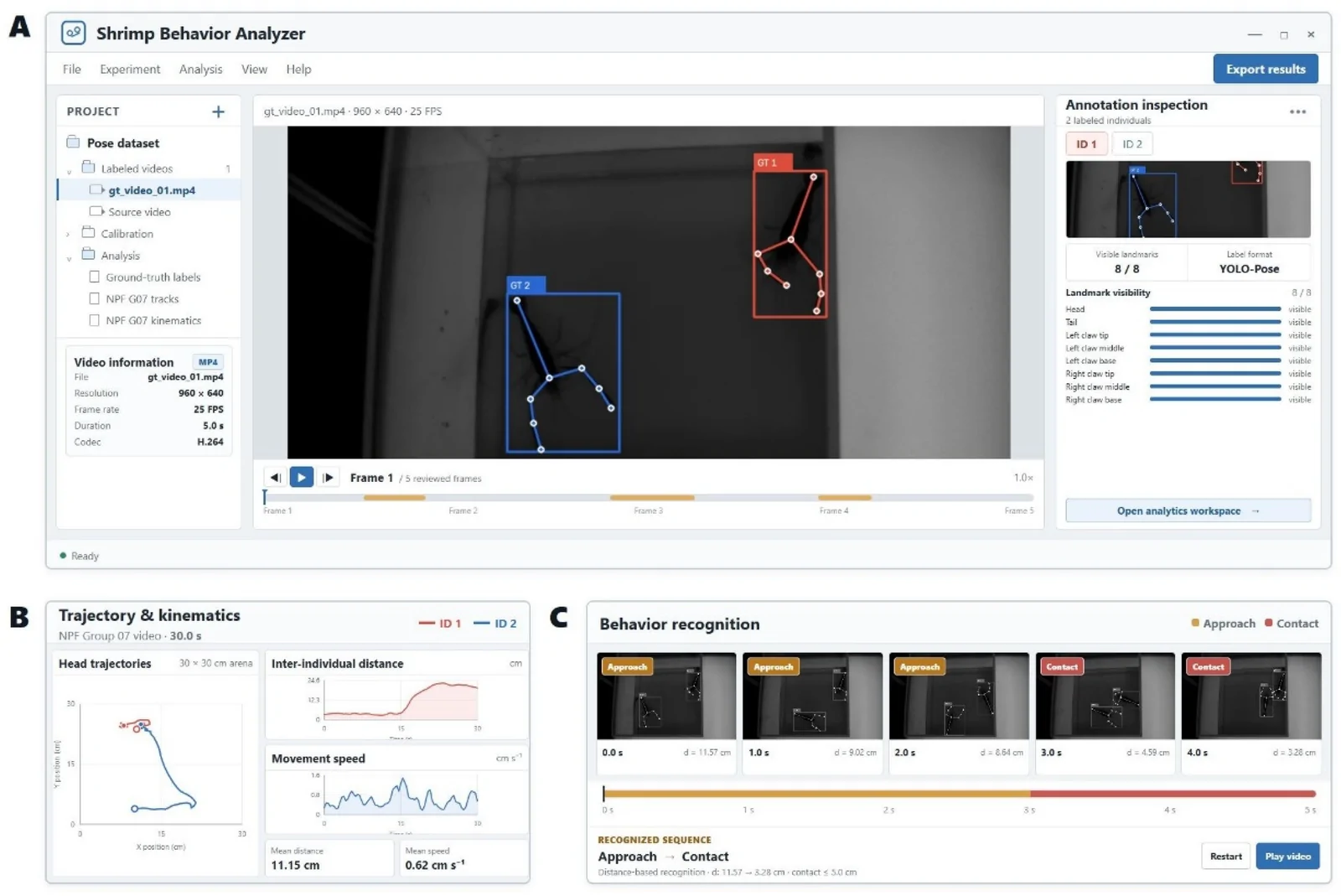
Software interface and representative outputs of the YOLOv11-Pose Tracking Platform. (**A**) Main workspace for project and video management, video playback, and visualization of identity-resolved detections and eight-keypoint poses. (**B**) Trajectory and kinematic analysis showing reconstructed head trajectories, inter-individual distance, and movement speed for a representative video segment. (**C**) Demonstration of the behavior segmentation function in the video playback module. For a behavior segment manually pre-determined and intercepted (showing a transition from approach to contact), continuous behavior can be segmented by setting custom thresholds. Red and blue represent the two tracked individuals; orange and red mark the segmented approach and contact sub-segments, respectively.

The software is a desktop-based visual interactive application developed with PySide6, integrating two core functions: tracking inference and quantitative behavior analysis. The video input module supports local multi-format files (e.g., MP4, AVI) as well as a USB live camera; the video loading interface is shown below (Figure 3, Initial software interface). The procedure for real-time online tracking is as follows:

i. ID initialization and keypoint visibility settings: After the first frame is loaded, the inference module outputs the two targets with the highest confidence. In the panel, the user can toggle the visibility state of the corresponding keypoints, and any unchecked keypoint will be excluded from all subsequent statistical analyses.

ii. Scale calibration: Before starting the tracking task, the calibration module must be invoked to complete the pixel-centimeter conversion. A standard-length reference object is used for two-point calibration to generate and save the exclusive conversion factor “r” for the current video. Formal behavior inference cannot be started without completing the calibration.

iii. Real-time tracking observation: Once inference is started, the rendering module simultaneously draws the prawn bounding boxes, currently visible keypoints, and target IDs. The interface outputs the real-time inference FPS, allowing full visual observation of the trajectories and posture changes of the two prawns throughout the process.

### 2.5. Measurement of Agonistic Behavior Regulated by NPF

Double-stranded RNA (dsRNA) specific to *NPF* and green fluorescent protein (GFP, as control) genes were synthesized using the T7 RiboMAX ExpressRNAi system (Promega, Fitchburg, WI, USA) kit as previously described [20,21,22]. The recombinant NPF protein without the N-terminal signaling peptide was expressed in *Escherichia coli* BL21 (DE3) (Invitrogen, Carlsbad, CA, USA) using a PET-32a(+) vector (Merck millipore, Darmstadt, Germany) containing the corresponding coding sequence of *NPF* gene following previously described procedures [23,24,25]. The recombinant Trx protein (as control) was expressed using empty pET-32a(+) vector. Proteins were purified using Ni-NTA agarose (Qiagen, Hilden, Germany), dialyzed in normal saline, and quantified using the BCA Quantification Kit. Both dsRNA and recombinant protein were administered via intramuscular injection at a dose of 1 μg/g body weight. Prior to injection, individuals in the control and experimental groups were randomly and equally distributed between two independent tanks for acclimation, ensuring that no inter-individual contact occurred before the experiment. At 48 h post-injection of dsRNA, qPCR was performed to verify the knockdown efficiency, and agonistic behavior assays were subsequently conducted with a 1 h observation period. For recombinant protein injection experiments, behavioral testing was initiated immediately after injection.

### 2.6. Effects of NPF on Growth, Feed Intake, and Glucose Metabolism

To evaluate the effects of NPF on growth, *M. rosenbergii* were injected with PBS (blank control), GFP- or NPF-specific dsRNA (dsGFP/dsNPF). Each group consisted of three biological replicates, with 15 individuals per replicate. Prior to the start of the experiment, initial body weight, abdominal length, and total body length were recorded for each group as baseline growth data, after which the first dsRNA injection was administered. Subsequently, dsRNA injections were repeated every 7 days for a total of four times, with growth measurements taken prior to each injection. Throughout the rearing period, excess feed was provided at fixed times twice daily (morning and evening), and uneaten feed was collected and removed 1 h after each feeding.

Feed intake was measured as follows. During the rearing period, all groups were fed with sufficient feed twice daily at fixed times (morning and evening). Prior to the measurement, feeding was withheld for 24 h to ensure a consistent fasting state across all groups. At the time of measurement, each group received 4 g of feed, which was evenly distributed over the bottom of the tank. One hour after feeding, the uneaten feed was collected using a siphon device, transferred to Petri dishes, dried to constant weight, and weighed. The actual feed intake was calculated as the difference between the amount offered and the amount remaining, and expressed in grams.

For the determination of energy metabolism-related enzymes, hepatopancreas tissues were homogenized in pre-chilled physiological saline at a ratio of 1:10 (g/mL) using a high-speed grinder at 2500 r/min. After centrifugation for 10 min, the supernatant was collected and appropriately diluted for subsequent assays. The activities of hexokinase (HK), phosphofructokinase (PFK), pyruvate kinase (PK), and glucose-6-phosphate dehydrogenase (G6PDH) activities, and NADP(H) levels were measured using commercial kits (Nanjing Jiancheng, China) following the manufacturer’s instructions [26].

### 2.7. Statistical Analysis

Differences among groups were evaluated by one-way analysis of variance (ANOVA), followed by Duncan’s multiple range test for post hoc comparisons. A significance level of *p* < 0.05 was considered statistically significant.

## 3. Results

### 3.1. Model Evaluation Metrics

To comprehensively evaluate the performance of the improved algorithm in underwater biological detection tasks, the following core evaluation metrics were selected: Precision (P), Recall (R), AP (Average Precision), PCK@10, MAEpx, and MAEcm.

Precision (P): Reflects the proportion of true positive samples among the samples predicted as positive by the model, measuring the model’s ability to avoid false detections. The formula is:

$$P=\frac{\mathrm{TP}}{\mathrm{TP}+\mathrm{FP}}$$

TP, the number of true positives; FP, the number of false positives.

Recall (R): Measures the model’s ability to detect all true positive samples, reflecting its capability to avoid missed detections. The formula is:

$$R=\frac{\mathrm{TP}}{\mathrm{TP}+\mathrm{FN}}$$

FN, the number of false negatives.

AP (Average Precision) represents the overall detection performance of the model under different confidence thresholds. The closer the value is to 1, the better the detection performance.

PCK@10 px is the proportion of predicted keypoints whose distance from the ground-truth keypoints is less than 10 pixels, relative to the total number of keypoints. A higher value indicates superior keypoint localization performance.

MAEpx (Mean Absolute Error in pixels) is the average absolute pixel error between the predicted coordinates and the ground-truth coordinates across all samples.

MAEcm (Mean Absolute Error in centimeters) is derived from the pixel error through a conversion factor, transforming the pixel error into an actual physical length error in centimeters.

### 3.2. Model Comparisons and Error Analysis

We compared pose estimation models from two architectural paradigms: CNN and ViT. Within the CNN paradigm, three scales of YOLOv11-Pose, Nano (n), Small (s), and Medium (m), were selected for error analysis, and the classic CNN baseline ResNet-50 was also included. Within the ViT paradigm, the lightweight ViT-pose-S model was chosen for cross-paradigm comparison. As shown in Table 2, for the YOLOv11-Pose series, AP and PCK@10px generally increased with network scale: the AP values of YOLOv11n-Pose, YOLOv11s-Pose, and YOLOv11m-Pose were 95.79%, 96.12%, and 96.55%, respectively, and their PCK@10px values were 77.42%, 78.86%, and 81.61%, respectively. Among them, the m-scale model achieved the best keypoint detection metrics, but its MAEpx (8.61 px) and MAEcm (0.366 cm) were both higher than those of the s-scale version. The n-scale model had the smallest number of parameters, with an MAEcm of 0.330 cm, while its PCK@10px was slightly lower than that of the s-scale. Considering detection AP, keypoint PCK@10px, and centimeter-level physical error, YOLOv11s-Pose achieved the best balance among the three, with superior physical-scale error performance.

Cross-paradigm comparison showed that the overall performance of ResNet-50 and ViT-pose-S was significantly weaker than that of the YOLOv11 series. ResNet-50 had an AP of only 77.26%, an MAEpx as high as 64.96 px, an MAEcm of 4.115 cm, and an inference speed of only 29.9 FPS; although its PCK@10px reached 79.08%, it exhibited clear shortcomings in detection accuracy, localization error, and inference speed. ViT-pose-S had an AP of 70.30%, a PCK@10px of 76.95%, an MAEpx of 74.42 px, and an MAEcm of 4.193 cm; its large keypoint localization error makes it difficult to meet the centimeter-level measurement requirements for behavioral quantification in *M. rosenbergii*. Considering detection accuracy, keypoint localization error, parameter count, computational cost, and inference speed across all models, YOLOv11s-Pose achieved the best overall performance among both CNN and ViT architectures. Combined with the per-keypoint validation results in Table 3, the subsequent software platform and behavioral quantitative analysis were both deployed based on the YOLOv11s-Pose weights.

When there are many predicted trajectories, substantial errors may accumulate. Therefore, a confidence rate (CR) is proposed to evaluate the analysis quality of the software observation. The specific formula is:

$$\mathrm{CR}=\frac{N_{\mathrm{total}}-N_{\mathrm{pred}}}{N_{\mathrm{total}}}\times 100\%$$

where *N_total_* represents the total number of frames in the inference portion of the video, and *N_pred_* is the number of frames completed by Kalman-filter predictions. A higher CR value indicates a higher proportion of frames with actual model detections in the video, lower accumulated trajectory errors, and thus higher reliability of the subsequent quantitative results for fighting and movement behaviors. In this experiment, the CR values of all videos ranged from 97.7% to 100%, with an average of 98.85%.

### 3.3. Regulation of Agonistic Behavior by NPF Gene

**Figure 4:**
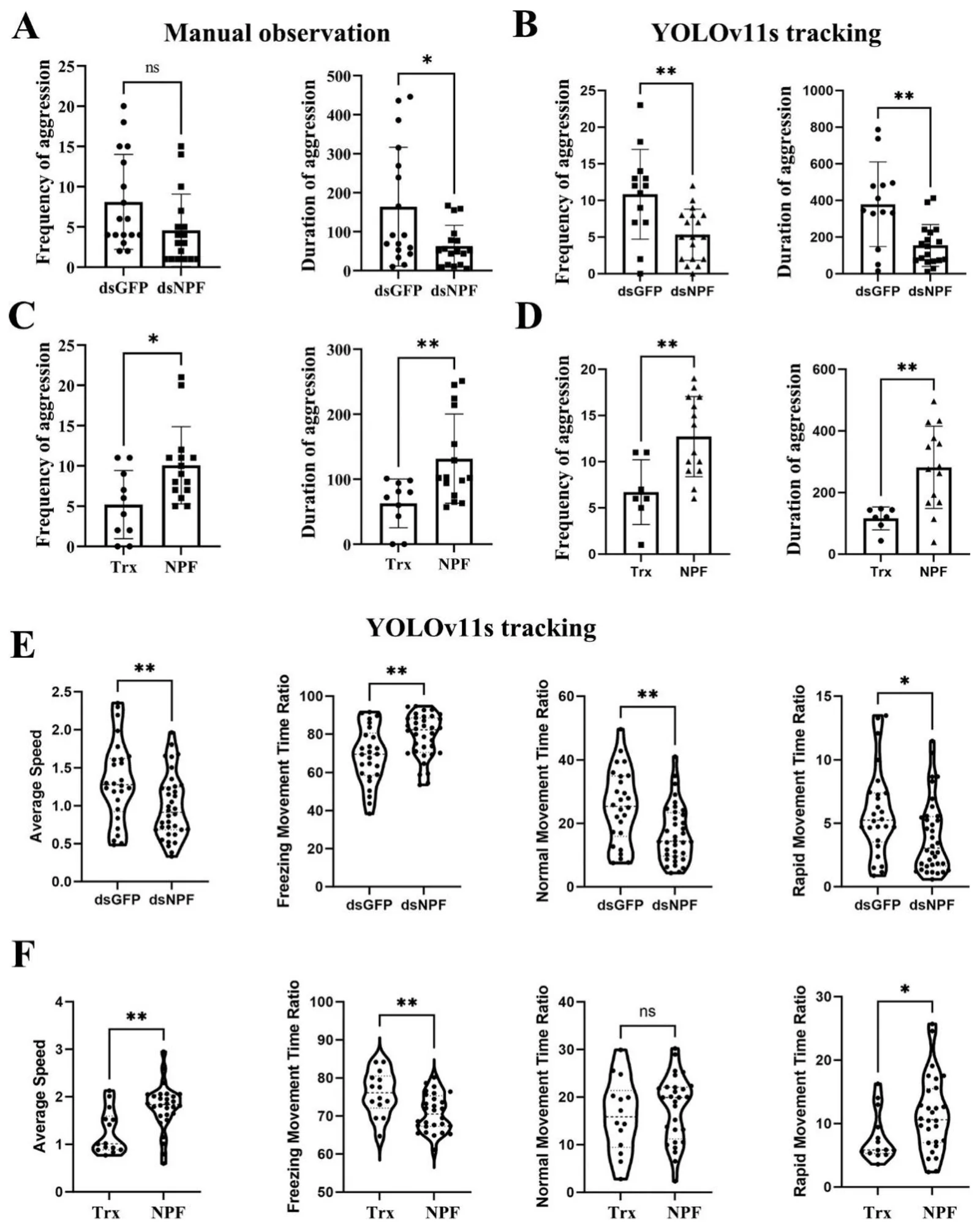
Regulation of agonistic behavior by NPF in *M. rosenbergii*. (**A**) Manual observation and (**B**) YOLOv11-Pose Tracking Platform-based quantification of fighting frequency (left) and duration (right) in NPF-knockdown prawns (dsNPF) versus dsGFP-injected controls. (**C**) Manual observation and (**D**) Platform-based quantification of fighting frequency (left) and duration (right) in recombinant NPF protein-treated prawns versus Trx-injected controls. The average speed, freezing movement time ratio, normal movement time ratio, and rapid movement time ratio in *NPF*-knockdown (**E**) and NPF protein-treated (**F**) prawns versus their controls. Each point represents the measured value of an individual prawn. Data are presented as mean ± SD. Each point represents the value for one prawn. Different symbol shapes (circles, triangles, or squares) are used to distinguish individual data points and do not denote groups; groups are indicated by the x-axis labels. *, *p* < 0.05; **, *p* < 0.01; ns, not significant.

NPF is a classic feeding-related molecule [27,28,29]. To validate the applicability and reliability of the established YOLOv11-pose tracking platform, we examined the regulatory effects of the *NPF* gene on agonistic behavior in *M. rosenbergii* and compared the outputs of manual observation with those generated by the automated platform (Figure 4). The role of NPF in agonistic behavior was further investigated through both loss- and gain-of-function experiments. The knockdown efficiency of the RNAi strategy targeting *NPF* was verified by comparing *NPF* mRNA levels in the ventral nerve cord, gill, intestine, and eye between prawns injected with *NPF*-specific dsRNA and those injected with GFP-dsRNA using qPCR (Supplementary Figure S1). Behavioral analysis showed that *NPF*-knockdown prawns exhibited reduced aggression frequency and duration compared to the controls (Figure 4A,B). Conversely, exogenous administration of recombinant NPF protein significantly increased aggression frequency and extended aggression duration (Figure 4C,D). Notably, these behavioral differences were consistently captured by both manual observation and the YOLOv11-pose-based tracking platform, with highly comparable quantitative outputs between the two methods. These reciprocal results collectively demonstrate that NPF functions as a positive regulator of agonistic behavior in *M. rosenbergii*, promoting both the initiation and the maintenance of aggressive interactions. Importantly, the consistency between manual and platform-based measurements validates the reliability and utility of the established automated system for behavioral quantification.

Furthermore, to supplement the measurement data on fighting behavior, the established platform yielded more kinematic parameters that are difficult to quantify accurately through manual observation, including average speed, freezing movement time ratio, normal movement time ratio, and rapid movement time ratio, thereby offering a more comprehensive quantitative dimension for behavioral assessment. Based on statistical analysis of the actual video data, the locomotor speed of *M. rosenbergii* was classified into three categories: freezing (<1.5 cm/s), normal movement (1.5–5 cm/s), and rapid movement (>5 cm/s). The threshold of 5 cm/s was derived from the extreme outlier boundary of the speed distribution, calculated as 3 × Interquartile Range (IQR) and rounded up to the nearest integer, serving to distinguish routine daily activities from high-intensity locomotor behaviors. The results showed that, compared to controls, *NPF*-silenced prawns exhibited a significant decrease in average movement speed, a marked increase in freezing movement time ratio, and significant reductions in both normal movement time ratio and rapid movement time ratio (Figure 4E). In contrast, individuals injected with recombinant NPF protein showed opposite trends in all these parameters (Figure 4F). These results indicate that NPF positively regulates locomotor activity in *M. rosenbergii*, and that alterations in its expression levels significantly affect movement intensity and behavioral vigor, which are associated with the degree of agonistic behavior. In other words, the NPF-mediated changes in locomotor capacity may represent a key behavioral basis through which this neuropeptide modulates fighting performance.

**Figure 5:**
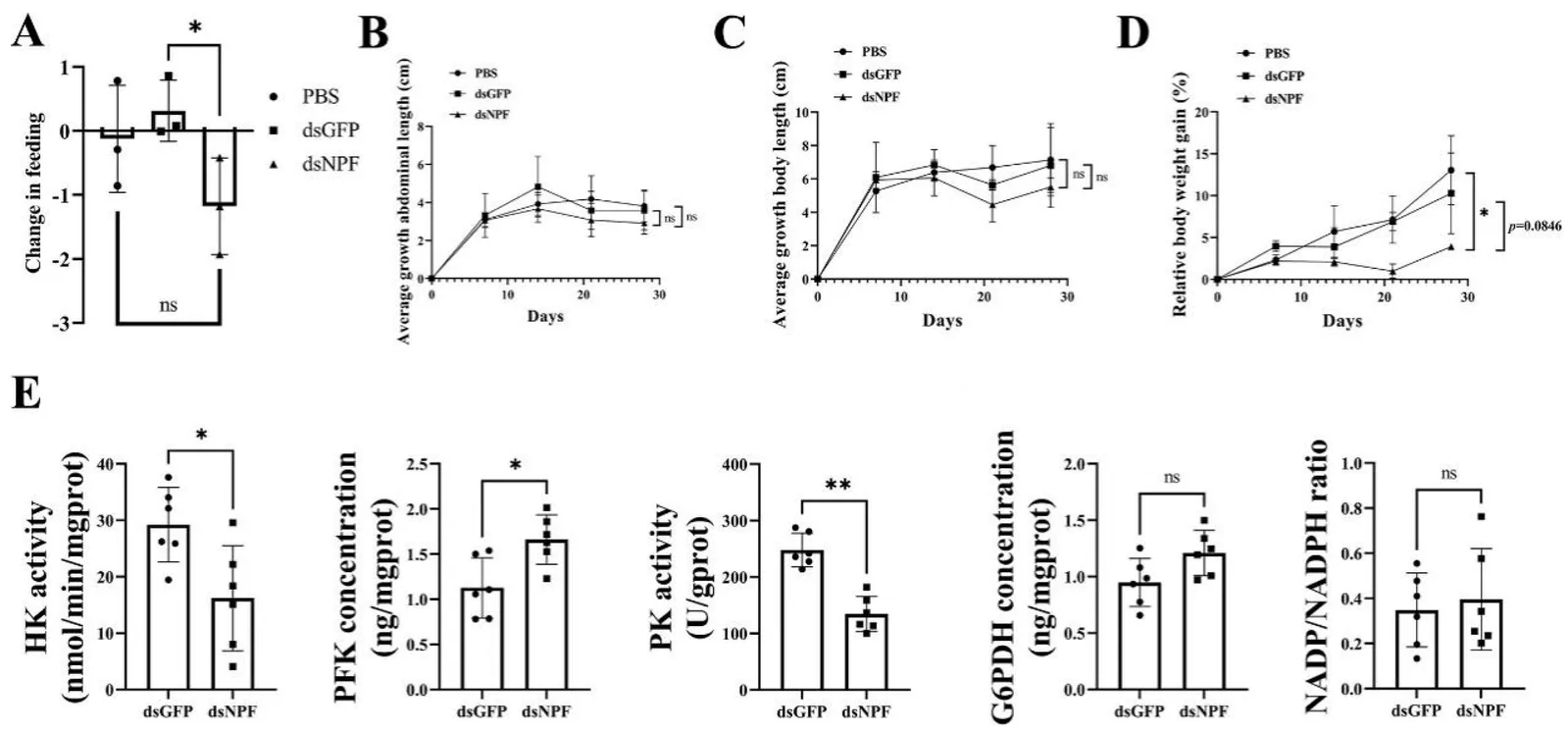
Effects of NPF on feeding, growth, and hepatopancreatic glucose metabolism in *M. rosenbergii*. (**A**) Feed intake at 48 h post-dsRNA injection in dsNPF, dsGFP, and PBS control groups (n = 15). Each point represents one replicate. (**B**–**D**) Growth performance over a 4-week period following dsRNA injection: abdominal length (**B**), body length (**C**), and body weight (**D**) in dsNPF, dsGFP, and PBS groups. (**E**) Activities of key glycolytic enzymes (HK, PFK, PK) and the pentose phosphate pathway enzyme (G6PDH), as well as the NADP/NADPH ratio in the hepatopancreas of *NPF*-knockdown prawns compared to controls. Data are presented as mean ± SD. *, *p* < 0.05; **, *p* < 0.01; ns, not significant.

In addition, to preliminarily explore the mechanisms by which NPF regulates agonistic behavior in *M. rosenbergii*, its effects on feeding, growth, and energy metabolism were examined. Compared to controls, *NPF*-knockdown prawns exhibited significantly reduced feed intake at 48 h post-injection (Figure 5A), confirming an orexigenic role for NPF in this species. For growth performance, we monitored abdominal length, body length, and body weight over a 4-week period following dsRNA injection (Figure 5B–D). Although the *NPF* knockdown group showed slightly lower abdominal and body lengths than the controls, these differences were not statistically significant. However, body weight in the knockdown group was markedly reduced compared to the PBS-injected group, despite the difference relative to the dsGFP-injected group not reaching statistical significance, suggesting that *NPF* knockdown exerts a detectable suppressive effect on growth. To further investigate the metabolic mechanism affected by NPF, the activities of key glycolytic enzymes and NADP/NADPH ratios in the hepatopancreas were measured (Figure 5E). The results demonstrated that in *NPF*-silenced prawns, HK activity was significantly decreased (*p* < 0.05), PFK activity was markedly increased (*p* < 0.05), and PK activity was sharply reduced (*p* < 0.01). By contrast, G6PDH activity and the NADP/NADPH ratio tended to increase, but the differences did not reach statistical significance. Collectively, these results indicate that *NPF* knockdown modulates feeding, growth, and hepatopancreatic glucose metabolism, suggesting that NPF may regulate agonistic behavior, at least in part, through its influence on energy metabolism.

## 4. Discussion

In this study, the YOLOv11-Pose series models were applied to estimate the body posture of *M. rosenbergii* and thereby evaluate various behavioral parameters. The model exhibited low training loss and pixel error, indicating excellent performance in predicting prawn posture. However, accuracy decreased under certain conditions, such as when the prawn adopted specific postures, when motion blur occurred, when manual annotation errors were present, or when body parts that were occluded and thus invisible—and not included in the training set—appeared in the images. These factors led to keypoint recognition errors and consequently prevented the network from identifying the target. Notably, expanding the training sample size through data augmentation reduced both loss and error and mitigated model inconsistency. A plausible explanation is that with an enlarged training set, the model was exposed to more unexpected postures from diverse angles, thereby improving its adaptability.

The loss- and gain-of-function experiments consistently demonstrated that NPF promotes agonistic behavior in *M. rosenbergii*, a role that contrasts with its aggression-suppressing function in *Drosophila*, suggesting evolutionary divergence in neuropeptide signaling between insects and crustaceans [30]. Importantly, all behavioral differences were captured equivalently by manual observation and the YOLOv11-pose-based platform, with highly comparable quantitative outputs across all treatments. This concordance validates the reliability and utility of our automated system for behavioral quantification, overcoming the subjectivity and low throughput inherent in manual scoring. Moreover, the software platform provided additional kinematic metrics—including average speed, freezing movement time ratio, normal movement time ratio, and rapid movement time ratio—that are not accessible through conventional manual scoring, thereby offering finer-grained insights into the behavioral effects of NPF manipulation. These parameters may reflect changes in energy expenditure, locomotion strategy, or motivational state, complementing the conventional frequency- and duration-based measures. Therefore, the established platform provides a robust, high-throughput tool for future behavioral screens in aquatic organisms.

Agonistic behavior in crustaceans is an energetically costly social activity, and its energy supply is highly dependent on metabolic pathways [31,32,33]. In the swimming crab *Portunus trituberculatus*, winners exhibited higher glycolytic and aerobic metabolic intensities than losers, indicating that glycolytic capacity directly influences fighting performance and outcomes [34]. Similarly, research on hermit crabs has demonstrated that individuals with higher hemolymph glucose concentrations are more aggressive and more likely to initiate and win fights [35]. The current study demonstrated that the positive regulation of aggression by NPF was also closely linked to its effects on energy metabolism. NPF knockdown induced a marked perturbation of glycolytic flux in the hepatopancreas, as evidenced by significantly decreased HK activity, increased PFK activity, and sharply reduced PK activity. This suggests that NPF deficiency disrupts the coordinated regulation of glycolysis, likely impairing the terminal conversion of phosphoenolpyruvate to pyruvate and thereby compromising ATP generation, the energy source for sustained aggressive interactions. In contrast, the pentose phosphate pathway remained largely unaffected, as indicated by insignificantly changed G6PDH activity and NADP/NADPH ratios. Given that the hepatopancreas serves as the central metabolic organ for energy storage and mobilization in crustaceans, the glycolytic disturbances induced by NPF knockdown in this study likely compromise the rapid energy supply required for fighting, thereby contributing to the reduced combat performance observed [36]. This supports the notion that NPF influences agonistic behavior, at least in part, through its modulatory effects on energy metabolism. Moreover, the glycolytic pathway may play a more critical role than the pentose phosphate pathway in fueling agonistic behavior in *M. rosenbergii*, possibly due to its capacity for rapid energy supply, which is better suited to meet the sudden and intense energy demands of fighting, a possibility that warrants further investigation [32,34]. Notably, NPF knockdown also significantly affected feeding and growth performance, suggesting that perturbations in energy metabolism may have pleiotropic physiological effects and implying a potential intrinsic link between growth and agonistic behavior [37]. It is also possible that fighting behavior represents a derived or modified form of foraging behavior [38]. These interconnected possibilities merit further experimental exploration and validation.

## 5. Conclusions

In this study, we developed a YOLOv11-pose-based automated tracking platform for quantifying agonistic behavior in *M. rosenbergii*. Validated through NPF loss- and gain-of-function experiments, the platform reliably captured behavioral differences comparable to manual observation while providing additional kinematic parameters. Mechanistically, NPF promotes aggression partly through modulating feeding, growth, and hepatopancreatic glycolysis, highlighting the role of energy metabolism in crustacean agonistic behavior. This approach offers a powerful tool for behavioral phenotyping in crustaceans. In addition, this study has several limitations. As noted above, although expanding the training set can alleviate some recognition issues, YOLOv11-Pose itself cannot distinguish active fighting from incidental physical contact, which is an inherent methodological limitation of using handcrafted geometric features for behavior recognition. Even with relatively strict fight criteria established based on extensive prior knowledge, the model can only ensure high consistency of keypoint outputs. Therefore, future work could incorporate additional handcrafted features or adopt video-based behavior classification to overcome the shortcomings of relying solely on object detection and pose estimation.

## Figures and Tables

**Table 1:** Configuration of the experimental environment.

Parameter Name	Value
Training epochs	200
Batch size	16
Image size	640 × 640
Initial learning rate	0.01
Learning rate momentum	0.937
Weight decay coefficient	0.0005
Optimizer	SGD

**Table 2:** Model comparison experiments for pose estimation of *M. rosenbergii*.

Model	P	R	AP	PCK@10px	MAEpx	MAEcm	Params/M	FLOPs/G	FPS
Yolov11n-pose	99.8	99.9	95.79	77.42	7.55	0.330	2.7	6.77	125.2
Yolov11s-pose	99.9	99.9	96.12	78.86	7.04	0.299	9.7	22.41	113.9
Yolov11m-pose	99.8	99.9	96.55	81.61	8.61	0.366	20.9	71.47	100.1
ResNet-50	83.51	83.7	77.26	79.08	64.96	4.115	28.0	163.34	29.9
ViT-pose-S	85.46	85.6	70.3	76.95	74.42	4.193	24.28	17.8	94.6

**Table 3:** Per-keypoint detection accuracy of the optimal YOLOv11s-Pose model.

Keypoint	PCK@10px	MAEpx	MAEcm
Head	84.40	5.47	0.241
Tail	89.54	4.85	0.218
Left Claw Tip	70.16	8.99	0.374
Left Claw Middle	81.01	6.42	0.282
Left Claw Root	80.07	6.44	0.270
Right Claw Tip	72.82	8.83	0.368
Right Claw Middle	77.78	7.78	0.330
Right Claw Root	73.53	7.83	0.324
Average	78.86	7.04	0.299

## Data Availability

The data that support the findings of this study are available from the corresponding author upon request.

## Supplementary Materials

The following supporting information can be downloaded at: https://www.mdpi.com/article/10.3390/ani16203307/s1, Figure S1: Knockdown efficiency of NPF gene by RNAi in gill, ventral nerve cord, intestine, and eye analyzed using qPCR. Data of the control dsRNA-GFP treated groups were set as the baseline (1.0). Each point represents the measured value of an individual prawn. Statistical analyses were performed using two-tailed unpaired Student’s *t*-test (* *p* < 0.05, ** *p* < 0.01).
